# Experiences and expectations regarding COVID-19 prevention and control measures among the hill tribe population of northern Thailand: a qualitative study

**DOI:** 10.1186/s12889-021-11145-5

**Published:** 2021-06-04

**Authors:** Siwarak Kitchanapaibul, Anusorn Udplong, Tawatchai Apidechkul, Ratipark Tamornpark, Thanatchaporn Mulikaburt, Peeradone Srichan, Soontaree Suratana, Fatima Yeemard, Pilasinee Wongnuch

**Affiliations:** 1 School of Health Sciences, Mae Fah Luang University, Chiang Rai, Thailand; 2 Center of Excellence for Hill Tribe Health Research, Mae Fah Luang University, Chiang Rai, Thailand

**Keywords:** COVID-19, Prevention and control, Experience, Expectation, Hill tribe

## Abstract

**Background:**

COVID-19 has been a major human threat for a year. A large number of people have been infected and killed globally, including hill tribe people living in remote and border areas between Thailand and Myanmar. Different expectations of and experiences with the implemented disease prevention and control measures by local, national and international organizations have been widely reported. This study aimed to understand the experiences and expectations regarding the disease prevention and control measures that were implemented among hill tribe people in Thailand.

**Methods:**

Qualitative data were collected from participants aged 20 and older who belonged to the hill tribes living on the border of northern Thailand and Myanmar. A semistructured questionnaire was used to guide interviews. Information was extracted for thematic analysis by the NVivo program.

**Results:**

Fifty-seven participants (36 female, 21 male) were interviewed; 27 participants were Thai Yai, 14 participants were Yunnan Chinese, 8 participants were Akha, and 8 participants were from other tribes. The average age was 45.8 years (min = 20 years, max = 90 years). Thirty participants had never attended school, and the other 27 participants had received education at different levels, from primary school to higher education. Forty participants were unemployed, 13 worked as agriculturists, and the other 4 were attending school. Both positive experiences, such as improving personal hygiene practices, maintaining close contact and increasing relationships among family members and demonstrating the leadership of the villager leaders, and negative experiences, including interruption of social interactions, family financial problems, poor access to medical care services, and invisible people to the government, were found. Different expectations were observed regarding organizations at the local, national, and international levels. Expectations at the local level included villagers and community leaders taking action to strongly contribute to prevention and control measures and to prevent unscreened people from entering the village. Obtaining accurate information about the disease and being financially supported were expectations at the national level, while closing borders to protect cases from overflowing into their villages was an international-level expectation.

**Conclusion:**

Although hill tribes reside in very remote rural areas, they experience both positive and negative effects of the disease prevention and control measures implemented by organizations. Their expectations are formally and informally voiced to policy makers at the local, national and international levels.

**Supplementary Information:**

The online version contains supplementary material available at 10.1186/s12889-021-11145-5.

## Introduction

COVID-19 has been globally recognized as a new and major threat to humankind. It has killed more than 2 million people, and more than 100 million people had been infected as of January 2021 [1]. The disease has had serious effects on many groups of people, such as elderly persons and those with chronic diseases such as diabetes [2], hypertension [2], and heart disease [3].

Many new cases have been reported among people who live in crowded areas and in close contact with each other [4, 5]. Given the rapid spread of the disease throughout the world, people in different countries face different levels of vulnerability to the disease [6, 7]. Public health measures have been introduced, such as maintaining distancing, wearing face masks and distance learning for schoolchildren [8, 9]. The pandemic has stimulated collaboration among people worldwide, particularly with respect to prevention and control measures [10, 11] at local, national, and international levels [12]. While various prevention and control measures have been implemented, many negative experiences have been reported for different groups of people who have different backgrounds in terms of education, economic status and citizenship [13].

Thailand was the first country to present a COVID-19 case after the first case was reported in China [14]. Throughout the rest of the year, Thailand introduced several disease prevention and control measures at both the national and local levels, such as staying at home, wearing face masks, and working from home [14–16]. The implemented measures were initiated by the ministry and the central government [15–17]. However, some measures are based on other major concerns, such as economic growth and education, and are unrelated to effective disease prevention and control [18–20]. Many interventions have been launched across the country, while others have been implemented in specific regions or areas; for example, Thailand has promoted traveling inside the country, and reduced taxes during specific periods of time [21]. Both positive and negative experiences have been observed in the implementation of prevention and control measures [22]. This means that some measures that have been implemented support economic growth but do not promote health, such as reduced support for airplane traveling and accommodation expenses during specific periods of time, which caused many people to remain in the same area. Some measures have had positive impacts on personal health but have negatively influenced the educational system [23, 24]. The impacts are experienced in different ways by different groups of people [25]. People with high economic and education levels might have negative experiences that differ from the experiences of people with poor economic status and low education, including people with stateless status.

The hill tribe people who live in the border areas of Thailand and Myanmar are among the stateless populations living in Thailand [26]. A hill tribe population of more than 4 million people lived in Thailand in 2020 [26, 27]. Approximately 30% of the hill tribe people in Thailand are not considered Thai citizens [26–28]. The Thai identification card, which presents a 13-digit number, is used for access to all public services, including access to medical care and educational systems [26]. During the COVID-19 pandemic, a large number of hill tribe people and other stateless populations were still living in the border areas of Thailand and Myanmar [27]. In late November, a cluster of 78 COVID-19 cases was reported through the surveillance system in Chiang Rai Province in the border area of Myanmar and Thailand [29]. As a result, many prevention and control measures were introduced to the people in these areas. Given the vulnerability of the hill tribe and other stateless populations living in these areas in terms of poor education, low economic status, low literacy and a lack of citizenship status, they might experience these measures in different ways.

This study aimed to understand the experiences and expectations of members of the hill tribes and other stateless populations living in the border areas of Thailand-Myanmar during the COVID-19 pandemic.

## Methods

A qualitative approach was used to understand the experiences and expectations of the disease prevention and control measures implemented among the hill tribe people in Thailand. Information was gathered from key informants living in 8 hill tribe villages located in the Thailand-Myanmar border area. The key informants included village health volunteers and villagers aged 20 and older.

Questions relevant to the topic were developed after discussion with people in the hill tribe villages about their experiences during the COVID-19 pandemic and their expectations of government agencies. All questions were pooled into a topic guide.

Two main issues were included in the study: experiences and expectations. First, six questions were used to extract information on the participants’ experiences regarding efforts to prevent and control the disease: a) Have you had any involvement in COVID-19 prevention and control? If so, how? b) How did you learn about COVID-19 prevention and control in your community? c) What did you do to protect yourself from becoming infected by the disease? d) How did you protect your family and community members? e) What are the best practices or measures that you have performed to prevent and control the disease? f) What are the negative impacts you have experienced from the prevention and control measures?

Second, four questions were used to collect information regarding the expectations of the hill tribe villagers: a) How do you expect government agencies to prevent and control the disease? b) How do you expect health officers to prevent and control the disease? c) How do you expect your community members to prevent and control the disease? d) How do you expect your family members to prevent and control the disease?

The set of topic guides was tested for quality by three experts in the field: an expert in social work, a public health professional, and an expert in behavioral science. The questionnaire was pilot tested with five hill tribe people living in Mae Chan District, Chiang Rai Province, who had characteristics similar to the study participants. All questions were reconsidered by the research team before their use in the field.

Seven selected village headmen were contacted 5 days prior to the interviews to verify essential information, including the characteristics of the key informants and the objectives of the study. On the date of data collection, which was in January 2021, the selected participants provided all essential information again, including the interviewees’ background and other information, to develop relationships before starting the interviews. Informed consent was requested from the participants, who signed the consent form if they agreed to take part. For participants who could not speak Thai, their fingerprints were obtained once they clearly understood the consent information, which was translated by village health volunteers fluent in both Thai and their language. The participants’ identities were protected through the use of pseudonyms.

Interviews were conducted at the participants’ homes using a face-to-face method. The interviews were taped with the approval of the participants, and field notes were taken. The interviews were conducted by four researchers (two males and two females) with extensive experience and knowledge in qualitative methods. During the interviews, the topic guide was used by the interviewer to determine course of the questions. Each interview lasted 40–50 min. Before the end of the interviews, the participants were asked to provide their mobile phone numbers in case additional information was required; however, no participants were contacted after the interviews because the information obtained was complete.

All taped interviews were transcribed into Thai. All documents were checked for errors again before being sent back to the participants for confirmation of the accuracy of the information. Coding trees of the main findings were developed, key findings were extracted, and data saturation was discussed among the researchers. All information was transferred into the NVivo program (NVivo, qualitative data analysis software; QSR International Pty Ltd., version 11, 2015) to derive the themes. Then, the findings were reconsidered by the research members before conclusions were drawn in the final step.

All research proposals, protocols, and tools were approved by the Human Research Ethical Consideration Committee from the Chiang Rai Public Health Province with IRB No. CRPPHO No. 72/2563. All methods were performed in accordance with the relevant guidelines and regulations. Before starting the interviews, all participants were provided with all relevant and essential information. Voluntary written informed consent was obtained from all participants before starting the interviews. Data were presented as extracted information with some quotations so that the overall information could not be used to identify any individuals. All information, including the records, was properly destroyed.

## Results

A total of 57 participants (36 female, 21 male) were interviewed: 27 participants were Thai Yai, 14 were Yunnan Chinese, 8 were Akha, and 8 were from other tribes. No participants refused the interview. The average age was 45.8 years, with a minimum of 20 years and a maximum of 90 years. The majority were married (63%), ever married (23%), and single (14%). Thirty (30) participants had never attended school, and the other 27 had received education at different levels, from primary education to higher education. Forty participants were unemployed, 13 worked as agriculturists, and the other 4 were attending school.

The findings are presented with regard to two major issues: experiences with the COVID-19 pandemic and the prevention and control measures and expectations regarding local, national, and international agencies. After thematic analysis, 34 codes under the two main coding trees or themes in experiences related to COVID-19 were found: positive and negative experiences. In the positive experience, three subthemes were detected: improving personal hygiene practices, maintaining close contact and increasing relationships among family members, and demonstrating the leadership of the villager leaders. Four subthemes were detected in negative experiences: the interruption of social interactions, family financial problems, poor access to medical care services, and invisible people to the government.

With regard to expectations of local, national and international agencies, three major subthemes were detected: expectations at the local, national and international levels. The hill tribe people expect all villagers, particularly community leaders, to follow prevention and control measures. With regard to their expectations at the national level, the hill tribe people expected to obtain financial support from the government, particularly during the period of lockdown implementation, and they required up-to-date information about the disease to apply to their daily life. People expected border ports to be closed to restrict human mobility, especially from neighboring countries where high cases of COVID-19 were reported.

### Experiences related to the COVID-19 pandemic and prevention and control measures

The hill tribe people had both positive and negative experiences with regard to the COVID-19 pandemic.

#### Positive experiences

In terms of positive experiences, many scenarios were identified, such as improving personal hygiene practices, maintaining close contact and increasing relationships among family members, and demonstrating the leadership of villager leaders.

##### Improving personal hygiene practices

Most of the hill tribe people in all age categories understood and accepted the practices of wearing face masks, regularly washing hands, using an alcohol gel, and maintaining social distancing. These behaviors were found to be widely practiced in public areas, such as in the market or supermarket and in medical institutions.

A 29-year-old woman said the following: [P#2]

“I always use a mask when going to the market. I think it is a good thing that all of us do to protect against the disease. I am not happy when I see someone not wearing a face mask when they enter the market. I feel we need to care for each other.”

A 45-year-old woman said the following: [P#9]

“I practice hand washing every day and use a mask, particularly when going out of my home. I feel much more comfortable talking with people who use masks, and I avoid speaking with people who do not wear masks.”

A 68-year-old man said the following: [P#37]

“It’s so surprising to me that everyone wears a mask and keeps distancing. I like it because my doctor told me that it will keep me safe from COVID-19.”

A 33-year-old woman said the following: [P#6]

“I myself am very strict with every one of my family members so that they keep wearing face masks and regularly wash their hands. I prepare a two-piece face mask for my two kids every morning before they go to school. I also prepare a small bottle of alcohol gel for them. Do you know, they do not go to school without these two things in the morning? They are now practicing automatically. I feel happy that everyone cooperates to save the others in my family by practicing using a face mask and regularly washing their hands.”

##### Maintaining close contact and increasing relationships among family members

The lockdown policy and restrictions on moving outside of the house and the village led the hill tribe people to spend more time with family members. People within the family joined in activities together over the months of lockdown. This situation had the greatest impact on elderly members of the family when their children returned home because many people had been working in large cities far from villages, such as Bangkok and Phuket.

A 29-year-old said the following: [P#33]

“I had been working in Phuket for 5 years with a good salary. After the lockdown policy, my company stopped running; then, I went back to my home for a while. Today, I have a lot of time to take care of my elderly parents. I think this is a good thing for me; I feel very happy. I cook several menus for my parents, which I never had time to do before.”

A 64-year-old woman said the following: [P#15]

“My two sons have been back in my family for three months since the company they work for stopped operating because of the lockdown policy. We have a good time and have many things to share with each other. They are starting to help me work on our farm. Because we are Yunnan Chinese, we have great family time during the season of Chinese New Year each year. We are now spending a lot of good time together.”

##### Demonstrating the leadership of the villager leaders

During the COVID-19 pandemic, people in villages were assigned both formal and informal tasks for prevention and control measures. The village headman had to handle almost everything for the community. Some villages experienced local quarantine and health checks, which required village members to collaborate and take action according to the protocol. Leadership was also widely observed among health professionals working as public health volunteers in communities.

A 48-year-old man said the following: [P#49]

“I am the village head in Moo 9 Terd Thai village. I have been assigned many things to implement by the district government officer since the first day the COVID-19 outbreak was reported in Thailand. I have to survey the list of the people who are at risk, I have to speak to my villagers about the disease every day, I have to meet with health professionals, I have to initiate the screening point at the entry port of the village. We need all village members to be involved in this program. I have to manage the village quarantine, which we use for 14 days to observe people who need to enter our village. I feel very tired, but I need to support the safety of my people.”

A 31-year-old woman said the following: [P#5]

“I am working as a village public health volunteer. I have been assigned by a doctor to schedule appointments for NCD patients and to care for two disabled people in the village during the COVID-19 pandemic. I have been asked to attend several conferences and meetings about COVID-19. I am also working as the spokesperson for COVID-19 in the village. I also joined the checkpoint and village guarantee programs. I am so proud of myself that I am able to help and save my people.”

A 40-year-old man said the following: [P#45]

“I am working as the community guard. I was assigned the work by the community headman. I did many things, such as investigating people who entered the village. We have to survey both in daytime and nighttime to prevent people from entering the village without being screened.”

Among the positive experiences of the hill tribe people during the severe COVID-19 pandemic, they seriously followed government recommendations and desired accurate and up-to-date information about the disease. Extensive information on death after infection and the lack of effective treatment pressured hill tribe people to follow government instructions. Moreover, because the residence area is a long distance from the city, accessing high-quality medical care, including vaccines, is not easy for them, so the best approach is to follow the instructions of the government. Following government instructions affected the hill tribe people through the limited treatment of the disease and limited power to negotiate with the government due to the lack of a Thai ID card. However, several positive experiences were identified in both personal and social adaptations.

#### Negative experiences

There were several types of negative experiences during the COVID-19 pandemic among the hill tribe population, such as the interruption of social interactions, family financial problems, poor access to medical services, invisible persons and lack of compensation from the government.

##### Interruption of social interactions

When the lockdown policy and social distancing measures were implemented, hill tribe social activities, including many religious rituals, were stopped or maintained with social distancing.

A 78-year-old man said the following: [P#51]

“I have not attended a temple for a while since the lockdown policy was implemented. Even today, we do not have a lockdown policy, but we still need to keep social distancing. I have spent a very short time at the temple for praying and shared fewer words with my friends. Basically, I do not like doing that and very much hope that it will disappear in the future. I need to have happiness while attending a temple.”

A 57-year-old woman said the following: [P#8]

“I had planned to hold a wedding ceremony for my daughter and her husband in the next couple weeks. However, the community health volunteer told me this morning that we should consider postponing the event for the next couple months because population movements are restricted, and large parties or entertainment events are not allowed by the government. We will follow the suggestion.”

A 37-year-old man said the following: [P#19]

“I am a village headman, and I received an unofficial message from the district government officer that the Chinese New Year ceremony will not be allowed this year. I am discussing with my team that if we need to proceed with the ceremony, we have to plan to quarantine the people who come to the village.”

##### Family financial problems

After COVID-19 emerged, many companies stopped operating because global logistics stopped, and product distribution chains were interrupted. Moreover, the measure limiting the number of people in one place affected the system of production. As a result, many people lost their jobs, including hill tribe people who worked at large companies in large cities. Almost every family faced new financial challenges.

A 31-year-old woman said the following: [P#27]

“I was laid off from my company after the COVID-19 pandemic began. I have two kids and parents who need to be supported. It is very sad that I cannot fully support them at the moment. I am now starting online selling of our farm products. My monthly income reduced from 50,000 baht per month to 4,000 baht. We have to save for family expenses, and we have started to grow many kinds of vegetables on our small land. I very much hope that the problem will be solved soon and I will able to go back to work at the company.”

A 42-year-old man said the following: [P#31]

“Before COVID-19 came, I worked as a motorcycle taxi driver and charged a little money for the service. However, nobody has come to this area since the disease emerged, and I lost my money. We have a big problem at the moment. None of my family members have the Thai ID card, so we do not meet the criteria to obtain compensation from the government. Those who hold the Thai ID card receive support from the government for at least one program. Unfortunately, we did not.”

A 58-year-old woman said the following: [P#42]

“I have a small shop and sell everything that needs to be used in daily life in this village and nearby villages. Since COVID-19 came, few people have come to buy stuff. I and my husband have a big problem with our family finances. We do not have our own land, and we never practiced farming. We pray every day that the problem will stop.”

A 20-year-old man said the following: [P#3]

“I am studying at a university in the city. I have a problem obtaining financial support from my parents. They told me that the money I get every month will be reduced and will cover only necessary items. I do not like it, but I have no better choice. I am planning to sell something online.”

##### Poor access to medical care services

During the pandemic, hospitals limited the number of patients who could access their services. Several approaches were implemented. Patients who had dental or oral problems, particularly those who were not severe or urgent, were told to postpone their appointments. Patients with chronic noncommunicable diseases were told to obtain medicine at a hospital near their home, and the time between visits to see a medical doctor was extended.

A 58-year-old woman said the following: [P#42]

“I have been informed by my dentist that the schedule for replacing my false teeth was postponed even though I have waited for more than 7 months. However, for the safety of everyone, I have no problem following the new schedule.”

A 65-year-old woman said the following: [P#18]

“I have been diagnosed with diabetes and hypertension for more than 10 years, and I was to see my doctor every month. However, when I saw my doctor in March 2020, he told me that all the next appointments were extended for three months. Although I am not very happy with the new schedule, I have no better choice. Luckily, I have not had any health problems during the three-month interval.”

A 48-year-old man said the following: [P#49]

“I have hypertension and need to see my doctor every month to check the status and receive medicine. I also have to take care of one of my sons, 16 years old, who has a severe mental health problem and needs to see his doctor to get medicines as well. The period of lockdown makes me worry very much. My son and I have to check our health regularly; if my son does not get his medicine, he presents bad emotions to me and everyone around.”

##### Invisible people to the government

According to a previous study, approximately 30% of the hill tribe people, particularly those who live in the border area of Thailand and Myanmar, have not been granted the Thai citizen card. This card is used for access to all public services and requests to obtain compensation from the government under the impact of many national policies, including the lockdown policy. Therefore, the voices and needs of those who do not hold a Thai ID card are consistently ignored by the government.

A 48-year-old man said the following: [P#29]

“I asked the government officers for support many times, but I did not get any response. All Thai people who hold Thai ID cards receive support from the government in some way. I was not granted a Thai ID card even though I have stayed in Thailand for more than 20 years. I know that I will not be supported or compensated by the government in any form, even though I am one of the victims of the COVID-19 pandemic and many of the national measures.”

A 64-year-old woman said the following: [P#15]

“I am 64 years old and have stayed in Thailand for more than 30 years, but I have not gotten a Thai ID card. I hoped to get the support of some money during the COVID-19 pandemic; unfortunately, I did not. I used my little money to buy stuff for a face mask and use it. I hope that I will get some support from the Thai government.”

Because the hill tribe people are a minority and lack an identity in Thailand, they faced severe family financial problems, particularly during the implementation of the lockdown policy. Due to the poor economic and educational background of the hill tribe and the lockdown, family finances have become a major problem for hill tribe people. While many people may be able to cope by growing vegetables on their farm, other people could not do so. The hill tribe people know that they have little power to request support from the government. Power is impossible for those who do not have Thai ID cards. The suffering is even worse for those who need medical care due to irregular schedules and the difficulty in finding help with transportation from home to the hospital and translation.

### Expectations regarding local, national and international agencies

During the COVID-19 crisis, many expectations were described by the hill tribe people living in the border areas of Thailand-Myanmar. These expectations can be classified into three levels: local, national, and international.

#### Expectations at the local level

Regarding local-level expectations, the hill tribe people asked the local people to take action to contribute to prevention and control measures. Community leaders acted according to government regulations to prevent unscreened people from entering villages, to maintain strict preventive and control measures such as masks, to maintain distancing among their people and to prevent community members from having social events, such as wedding ceremonies and new house ceremonies.

A 42-year-old man said the following: [P#31]

“I am one of the village guards, and we have been assigned many things to do to protect our village members from COVID-19. I hope the actions that we are doing will continue until COVID-19 disappears. I think it is very important to all of us.”

A 34-year-old woman said the following: [P#46]

“As a village public health volunteer, I very happy to do many things every day to save our people. I think the activities that we are doing are very useful to our village members. I very much hope that the public health office will support us in doing these actions continuously until the time when we truly do not need them.”

A 47-year-old women said the following: [P#58]

“I have been back in my village for three months, but before I could enter my house, I went through several steps of the screening and spent 14 days in the village quarantine areas. I like it because it keeps us safe from the disease.”

#### Expectations at the national level

At the national level, the hill tribe people wanted updated information regarding COVID-19 from health professionals. They also wanted to obtain support, particularly financial support, for their family from the government, which was truly needed during the lockdown. Many people hoped to receive a vaccine for COVID-19.

A 73-year-old man said the following: [P#11]

“I am looking for the vaccine. I think the government and the staff from the Ministry of Public Health should hurry in providing the vaccine to us. I believe that if we all get the vaccine, we can do everything we did in the past.”

A 42-year-old man said the following: [P#31]

“I think I should get financial support from the government. I just met the village headman a couple days ago to ask about my request to get some money from the government. This is very important to me. My family is now having a big problem with no money to use in our daily life. I have three children and two elderly parents that I have to support. I have borrowed some money from two sources over the past eight months. I also need a job now.”

#### Expectations at the international level

Regarding international-level expectations, the hill tribe people supported the country’s policy to close the border with Myanmar. The hill tribe villages are connected with Myanmar, and a number of people cross the border every day in normal situations. However, most hill tribe people expressed their support for the border closure policy until COVID-19 was properly addressed.

A 48-year-old man said the following: [P#29]

“As the village headman, I think closing the border is very important to stop the disease from spreading to my population. I support this policy.”

A 58-year-old woman said the following: [P#42]

“I sell many things to people through my own small grocery shop. In the past, many Burmese came and bought things at my shop. However, I think closing the border is very good; I support this even if I cannot sell stuff as well as usual.”

The hill tribe people expected to be safe from the disease and to receive financial support. They needed good collaboration from their villagers to cooperate with the prevention and control measures implemented. They also expected to obtain up-to-date information, particularly on how to protect themselves and their family members. Because their residences are located in border areas, they also expected to stop people from crossing the borders, particularly people from high incident case countries. All the hill tribe people expressed their expectations under the threatening circumstances.

In summary, the hill tribe people had both positive and negative experiences. Three levels of expectations during the COVID-19 pandemic were identified (Table 1).

**Table 1:** Impacts and experiences identified by participants for community subpopulations

Category	Positive experiences	Negative experiences	Expectations
***Children***	- Spending time with their parents and family members -Improved personal hygiene practices	-Difficulty accessing distance learning	-Receiving financial support from the government -People, including all leaders, should be strongly involved in prevention and control measures to protect people from infection -People expect to receive a vaccine -People need to protect the immigrant population in this time, particularly people from high incident areas such as Myanmar
***Women***	-Having a closer relationship among family members	-Losing jobs and facing family finance problems -Working difficult and harder jobs on the farm to maintain the source of family food - Less opportunity to visit and care for older parents	
***Working population***	- Spending time with family members -The opportunity to protect community members from the disease through involvement in prevention and control implementation	-Losing jobs and having financial problems -Losing the opportunity to join community and religious activities - Having family financial problems -Losing the opportunity to obtain compensation from the government for those who have not been granted Thai ID cards	
***Elderly people***	- Maintaining close relationships with family members who live together	-Losing the opportunity to join community and religious activities. - Difficulty accessing medical care -Some people have no offspring to care for them during this difficult time	

## Discussion

Fifty-seven hill tribe people with different demographic characteristics provided information about their experiences during the COVID-19 pandemic and the implementation of prevention and control measures by the government. Both positive and negative experiences were reported. The positive experiences reflected the improvement in personal hygiene, in family relationships, and in the leadership of the village leaders. Negative experiences included the interruption of social interaction, financial family problems, poor access to the medical care system, and noncompensation from the government. However, many expectations were proposed formally and informally regarding organizations at the local, national, and international levels.

The hill tribe people described many positive experiences during the COVID-19 crisis, such as improved personal hygiene skills, improved family relationships, and improved leadership skills among community leaders. This finding coincides with a study conducted in Israel [30], which reported that during the COVID-19 pandemic, the population dramatically improved their skills in and frequency of handwashing and using masks. A study in a hospital [31] found that people working in the hospital significantly improved their personal hygiene, particularly hand washing, during the COVID-19 pandemic. Thailand has launched a new normal lifestyle to improve people’s COVID-19 behaviors, such as maintaining social distancing, regularly washing hands, and using a mask, which greatly impacts people’s health behaviors [32]. Due to the promotion of the new normal lifestyle and COVID-19 infection, the hill tribe people strongly adhere to these measures.

The hill tribe people reported that the COVID-19 pandemic allowed them to spend time with their family members. As a result, family members developed strong relationships. A study in China clearly demonstrated that spending a long time with family members could significantly improve the family relationship [33]. However, Zhang [34] reported that when family members in China spent extensive time together during the COVID-19 pandemic, family violence increased. A study in Thailand reported that under the lockdown policy [35], the time family members spent together improved their relationships and effectively enhanced their collective power to face problems. Therefore, the development of better family relationships during the long lockdown in Thailand is one of the advantages the hill tribe people have gained.

Moreover, some hill tribe people reported that during the COVID-19 pandemic, many community leaders demonstrated their leadership to prevent and control the disease through programs and activities. This was supported by the United Nations High Commissioner for Refugees (UNHCR), which reported that community members and community leaders, particularly those living in remote areas, were the key persons in the response to the COVID-19 pandemic through their leadership skills [36]. Mehta et al. [37] reported that many leadership skills emerged among community leaders in India that were crucial for successful COVID-19 prevention and control. This could be one of the best opportunities to improve leadership skills among hill tribe village members and leaders.

Several negative impacts were also found among the hill tribes during the COVID-19 pandemic, such as limited socialization among community members, family financial problems, and poor access to medical services. Hill tribe people usually live in small villages and regularly engage in social interactions in different forms, such as wedding ceremonies and new house ceremonies. However, during COVID-19, no social events were allowed, particularly crowded interactions. Several guidelines have been developed to maintain social distancing during the pandemic [38–40]. However, to the best of our knowledge, no study has investigated the impact on social events in a community and people’s lifestyle from the perspective of the policy of social distancing. In our study, the prohibition of social interaction led to suffering and impacted the communal lifestyle of the hill tribe community in Thailand.

In this study, various expectations of individuals and organizations from hill tribes living in remote border areas were found at different levels. At the local level, the people wanted to see community leaders perform their roles in the prevention and control measures implemented, and they wanted to see their fellow community members maintain behaviors such as using face masks, maintaining social distancing, and washing hands. This reflects strong adherence to the government’s health recommendations to prevent and control the disease. A report from the United Kingdom observed that several COVID-19 prevention and control measures were effective when the community paid attention to and engaged in the program [41]. Moreover, a report from the United States observed that the collaboration and investment of the local government was key for successful COVID-19 prevention and control in certain areas [42]. A small study in Thailand reported that collaboration between the government and the community, especially community leaders, in performing their roles under the guidelines was a success factor in controlling the disease in urban areas [43]. With few options or strategies to prevent and control COVID-19, hill tribes need collaboration among community members, particularly community leaders, to oversee local quarantines and screening points before entry into villages.

This study found that the expectations of the hill tribe people at the national level included the desire to receive updates and accurate information regarding COVID-19 and financial assistance from the government, while some people expected to receive the COVID-19 vaccine. This finding coincides with a study in Wuhan, China, which reported that effective risk communication was one of the aspects desired by the people living a community [44]. Aslani [45] reported that during the crisis of the COVID-19 pandemic, people sought accurate and updated information. The World Health Organization [46] also reported that people in Thailand sought accurate and updated information on COVID-19.

In terms of financial support, hill tribes need to receive financial support. This coincides with a report from the International Labor Organization (ILO) stating that people need to obtain financial support from the government, particularly in lockdown situations [47]. The government of Thailand implemented a new policy of financial support for those who are highly vulnerable to economic problems [48]; however, its implementation has not been advantageous to hill tribe people who do not hold Thai ID cards [26]. As another expectation of organizations at the national level, the hill tribe members expected to receive the COVID-19 vaccine; unfortunately, Thailand has not yet implemented a vaccine.

All the hill tribe villages are located in the border areas of Thailand and Myanmar. Given the serious pandemic in Myanmar [49], the hill tribe people living in the border area are at risk of COVID-19. Therefore, they expected to obtain help from international organizations to protect them from people across the border.

One limitation was found in this study. Some participants were not fluent in Thai; in such cases, local public health volunteers were asked to help translate during the interview. As a result, some content may not have been fully obtained from the participants. However, before the analysis was performed, the typed contents were sent to the interviewee for confirmation or correction.

## Conclusion

During the global COVID-19 pandemic, difficulties in both the health and economic spheres have been encountered by everyone, including the hill tribe people living in remote rural areas. Given the conditions of their residence, they are at risk of disease infection because they live in the border areas of Thailand and Myanmar and are reported to face one of the highest infection levels in Southeast Asia. This study described the positive and negative experiences of hill tribe people during the pandemic and the impacts of the prevention and control measures implemented by the Thai government. The people’s expectations of policy makers at the local, national and international levels were also outlined. Because they face poor economic conditions and education and live in high-risk areas, the hill tribe people have had different experiences and have voiced their expectations both formally and informally.

Recommendations can be proposed as follows: i) the government should implement a suitable program to support or address financial problems, such as microeconomic programs, to these populations that do not require Thai citizenship; ii) a mobile clinic should be implemented to care for people at the village level, which could reduce the exposure time and the opportunity for the spread of infection; and iii) a specific learning program should be developed for students that is less dependent on technology and costly approaches; this program could involve a small learning group in a village.

## Supplementary Information

**Additional file 1.** Topic guide.

## Data Availability

The datasets used in the study are available from the corresponding author upon reasonable request.

## Abbreviations

COVID-19: Coronavirus Disease-2019
ID: Identification card
ILO: The International Labor Organization
IRB: Institutional Review Board
UNHCR: The United Nations High Commissioner for Refugees
WHO: World Health Organization
