# Supplementary material for: Experiences and expectations regarding COVID-19 prevention and control measures among the hill tribe population of northern Thailand: a qualitative study
Source: BMC Public Health. 2021 Jun 4;21:1060. doi: 10.1186/s12889-021-11145-5 (PMC8176874; doi:10.1186/s12889-021-11145-5)
Supplement: Supplementary file 1 — Additional file 1. Topic guide. [file 12889_2021_11145_MOESM1_ESM.docx]

**Questions guideline of the interview**

1. The experiences of prevention and control covid-19.

a) Have you had any involvement in COVID-19 prevention and control? If so, how?

b) How did you learn from COVID-19 prevention and control in your community?

c) How did you do to protect yourself from becoming infected by the disease? d) How did you do to protect your family and community members?

e) What are the best practices or measures that you have led to prevent and control the disease?

f) What are the negative impacts you have experienced with the prevention and control measures?

1. The expectations of the hill tribe villagers.
2. How do you expect to the government agencies to prevent and control the disease?
3. How do you expect to the health officers to prevent and control the disease?
4. How do you expect to your community member to prevent and control the disease?
5. How do you expect to your family member to prevent and control the disease?
